# Extracellular matrix remodeling in the tumor immunity

**DOI:** 10.3389/fimmu.2023.1340634

**Published:** 2024-01-25

**Authors:** Wei Du, Xueming Xia, Fan Hu, Jiayun Yu

**Affiliations:** ^1^ Department of Targeting Therapy and Immunology, West China Hospital, Sichuan University, Chengdu, China; ^2^ Division of Head & Neck Tumor Multimodality Treatment, Cancer Center, West China Hospital, Sichuan University, Chengdu, China; ^3^ Key Laboratory of Birth Defects and Related Diseases of Women and Children, West China Second University Hospital, Sichuan University, Chengdu, China; ^4^ Department of Radiation Oncology, Cancer Center, West China Hospital, Sichuan University, Chengdu, China

**Keywords:** extracellular matrix, tumor microenvironment, stiffness, cancer, immune cells

## Abstract

The extracellular matrix (ECM) is a significant constituent of tumors, fulfilling various essential functions such as providing mechanical support, influencing the microenvironment, and serving as a reservoir for signaling molecules. The abundance and degree of cross-linking of ECM components are critical determinants of tissue stiffness. In the process of tumorigenesis, the interaction between ECM and immune cells within the tumor microenvironment (TME) frequently leads to ECM stiffness, thereby disrupting normal mechanotransduction and promoting malignant progression. Therefore, acquiring a thorough comprehension of the dysregulation of ECM within the TME would significantly aid in the identification of potential therapeutic targets for cancer treatment. In this regard, we have compiled a comprehensive summary encompassing the following aspects: (1) the principal components of ECM and their roles in malignant conditions; (2) the intricate interaction between ECM and immune cells within the TME; and (3) the pivotal regulators governing the onco-immune response in ECM.

## Introduction

The extracellular matrix (ECM) is a complex tridimensional molecular framework that surrounds and supports cells within tissues. This intricate structure consists of a diverse range of macromolecules, including, but not limited to, proteins such as collagens, proteoglycans (PGs), and matricellular proteins, as well as glycosaminoglycans (GAGs) like hyaluronan (1). The ECM plays a crucial role in tissue development, maintenance of homeostasis, and the regulation of disease processes. It achieves these functions through its provision of structural support, control of cellular activities, and facilitation of interactions between cells and the matrix. The various components of the ECM engage in reciprocal interactions with one another and with cellular entities through specific binding sites. This interaction is essential for the proper structural organization and functional integrity of the ECM scaffold (2).

The importance of the physical properties of the ECM, specifically its stiffness, is increasingly supported by recent research, particularly in relation to the tumor microenvironment (TME). Unlike the ECM’s durable proteins, the immune cells in the TME display a dynamic behavior. The ECM, which is characterized by collagen crosslinking and the presence of glycoprotein-mediated bioactivators, plays a vital role in transmitting signals that govern the functions of immune cells. Despite the extensive examination of these aspects in previous literature (3–5), the interaction between the ECM and immune cells remains an area that has received relatively limited attention. The objective of this review is to comprehensively investigate the relationship between ECM stiffness, its components, and their influence on immune cells.

## An overview of the ECM in TME

The ECM is an intricate assemblage of proteins, glycoproteins, and polysaccharides that envelops cells within tissues. In addition to furnishing structural reinforcement, the ECM also governs diverse cellular mechanisms such as growth, proliferation, and organoid formation (6). Research has demonstrated that the mechanical characteristics of the ECM, encompassing its elasticity and viscoelasticity, exert an influence on cellular behavior and may have significant ramifications in the realms of tissue development, homeostasis, regenerative processes, and disease progression (7, 8).

From the spatial perspective, the ECM can be classified into two primary types, distinguished by their composition and localization. The interstitial connective tissue matrix represents one such type, encompassing cells and offering a structural framework for tissues. This particular ECM type supports the underlying stromal compartment and peri-cellular membrane. The second type is known as the basement membrane, serving to separate the epithelium from the adjacent stroma and support epithelial/endothelial cells (2). The advancement of invasive cancer is contingent upon the degradation of the adjacent ECM, a phenomenon that not only promotes cancer proliferation but also results in the deterioration of healthy tissue. Notably, this degradation of the ECM is accompanied by the deposition of a unique tumor-specific ECM, which typically demonstrates heightened density and stiffness (9–11).

### Basement membrane

The basement membrane (BM) is strategically positioned at the interface of parenchymal and connective tissues. Its principal role is to establish a supportive, sheet-like structure that maintains the integrity of parenchymal cells and inhibits their separation (12, 13). In the context of epithelial cancers, the BM plays a crucial role as a protective barrier against the infiltration, dissemination, and migration of cancer cells. Changes in the BM are commonly observed during the progression of cancers. Cancer cells typically breach the BM through various mechanisms, including the secretion of enzymes that remodel the extracellular matrix, exploiting existing pores in the BM, or applying mechanical force to penetrate these pores (14). In areas where laminins, fibronectin (FN), and collagen types I, III, and IV are arranged in sheet-like formations, the BM undergoes significant thickening. This architectural configuration effectively segregates the tumor into cancer cell nests and stromal regions, thereby facilitating the malignant attributes of cancer cells and influencing the modulation of the immune response in the TME (15–17).

### Interstitial matrix

In a physiologically sound environment, the interstitial matrix (IM) is characterized by a loosely organized extracellular matrix that closely interacts with the basement membrane. This matrix is composed of a combination of collagen types I and III, as well as elastin fibers and glycoproteins. Within this framework, fibroblasts, resident immune cells, and a complex network of blood vessels and lymphatics can be observed (18, 19). In certain tumors, the collagen fibers within iIM exhibit greater thickness, enhanced organization, and increased density as a result of heightened collagen deposition (17, 20). As cancers progress, the stromal collagen fibers gradually align themselves, particularly at the periphery of the tumors, thereby facilitating the invasive behavior of cancer cells (21, 22). Moreover, the lysyl oxidase (LOX) family, a crucial enzyme group, plays a vital role in the facilitation of collagen cross-linking (23). In the context of tumor scenarios, an elevation in LOX expression results in an excessive cross-linking of collagen. Consequently, this phenomenon is accompanied by an augmentation in collagen deposition, leading to a substantial enhancement in the stiffness of the tumor. Consequently, solid stress is induced within the tumor (24). Alongside changes in collagen, various components within the ECM also demonstrate atypical expression.

In essence, the ECM in TME not only offers mechanical support to cells, ensuring their structural integrity, but also functions as a reservoir for growth factors, cytokines, and other signaling molecules. It possesses the ability to sequester these molecules and subsequently release them in a regulated manner, thereby exerting control over cellular behavior (7). Moreover, the ECM can modulate the activity of growth factors by binding to them and influencing their availability and stability (25). This highly dynamic structure undergoes continuous remodeling through enzymatic and non-enzymatic posttranslational modifications, which in turn modify its instructive capacity (7).

The abnormal ECM can exert various influences on immune cells. Specifically, the stiffness of the ECM can directly impede the migration of immune cells. When the ECM undergoes abnormal stiffening, it can function as a physical obstacle, impeding the movement of immune cells. Certain immune cells, including endothelial and immune cells, possess the ability to generate matrix metalloproteinases (MMPs), which degrade the ECM at the leading edge, thereby establishing localized pathways that facilitate unrestricted migration of immune cells. Meanwhile, tissues inhibitors of MMPs (TIMPs) are produced to balance between matrix degradation and production. These ongoing interactions between the ECM and diverse immune cells involve dynamic and reciprocal biochemical and biomechanical dialogues that persist over an extended period of time.

## The main matrix components

**Figure 1:**
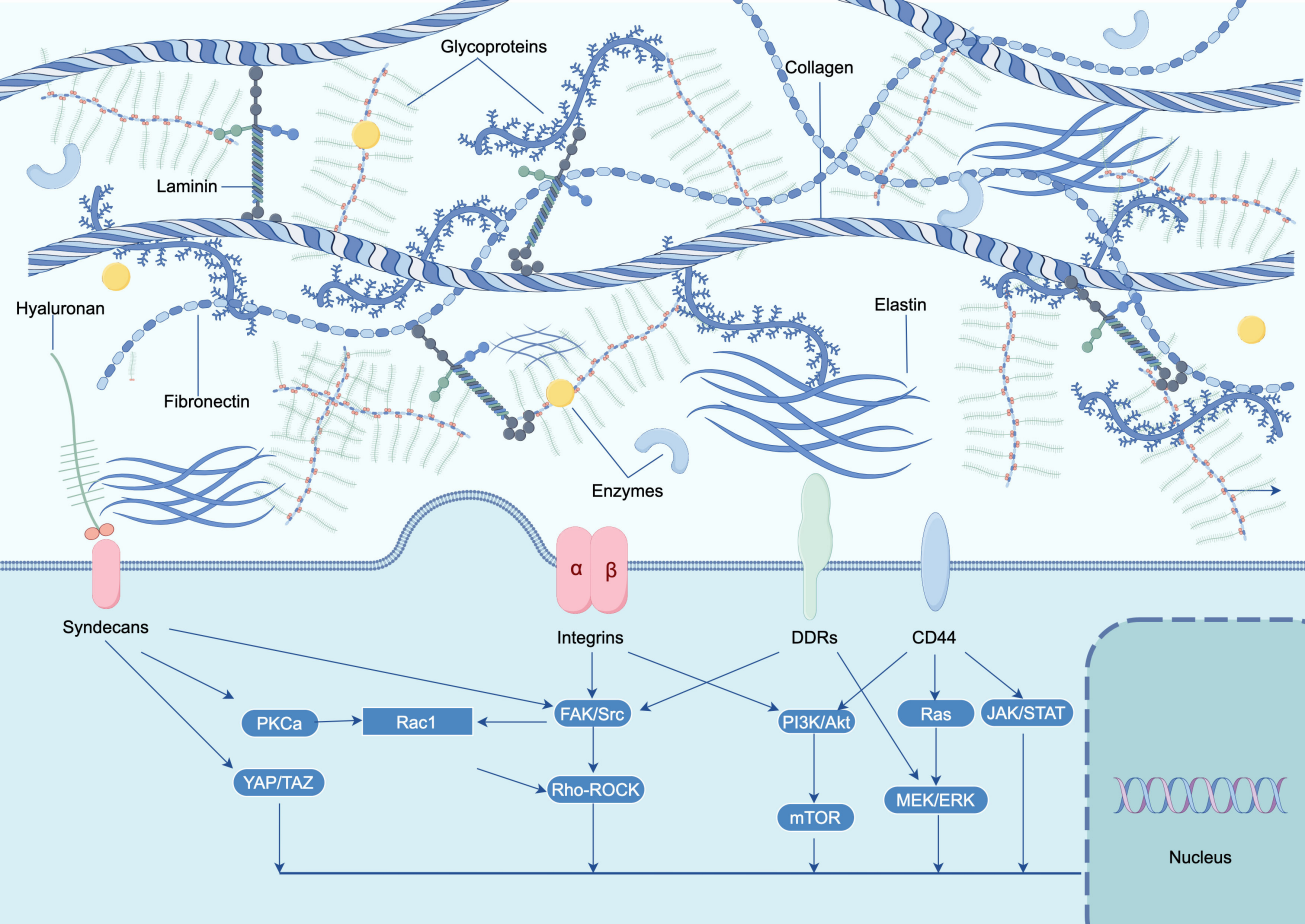
The modulation of intracellular signaling in TME through alterations of the ECM. The ECM changes play a significant role in influencing various intracellular signaling pathways. The primary mediators of extracellular cues are integrins, which integrate biochemical and biomechanical cues with growth factor signaling and other inputs. Additionally, receptors such as epithelial DDRs, syndecans, and the hyaluronan receptor CD44 transduce matrix signals. Ultimately, these signals regulate multiple cellular functions, including cellular adhesion, cytoskeletal dynamics, cell proliferation, cell survival and differentiation. ECM, extracellular matrix; TME, tumor micro environment; FAK, focal adhesion kinase; GAG, glycosaminoglycan; ROCK, Rho-associated protein kinase; YAP/TAZ, yes-associated protein/transcriptional co-activator with PDZ-binding motif.

The presence of distinct classes of macromolecules within the ECM of various tissues facilitates the specialization and diversity of tissue functions (3, 4) (**Figure 1**). The synthesis of diverse components within the ECM is governed by two factors: the stimulus and the temporal and spatial parameters of stimulation. These factors determine the range of components that cells can produce within the ECM, thereby influencing the magnitude of the cellular response (26). Variations in composition, cellular binding sites, and associated proteins contribute to the distinct structural and biological properties observed in basement membranes across different locations (5, 27).

Collagens, as the most prevalent protein in mammals, constitute approximately 30% of the overall protein mass. Functioning as a multidomain protein, collagens hold significant importance in maintaining the structural integrity and mechanical attributes of tissues. Comprised of three polypeptide chains, referred to as alpha chains, collagens can form either homotrimers, where the alpha chains are identical, or heterotrimers, where the alpha chains differ. The classification of collagens into distinct subfamilies is based on their supramolecular assemblies, including fibrils, beaded filaments, anchoring fibrils, and networks (28) (**Figure 1**). A total of 28 distinct collagens, designated numerically as I-XXVIII and some possessing colloquial names, have been identified in vertebrates (29). It is important to note that not all of these collagens exhibit protumorigenic properties. The precise combination and proportion of these various collagens play a crucial role in determining the compliance, stiffness, porosity, as well as the viscoelastic and biochemical characteristics of the extracellular matrix in both tissue homeostasis and cancer. Collagen I, the most prevalent type of fibrillar collagen in human tissue, is predominantly present in connective tissues such as skin, bone, tendon, and cornea. It forms fibrous structures that offer essential structural support and mechanical strength to these tissues. The composition of collagen I involves a right-handed triple helix resulting from the twisting of left-handed helical α chains. Each collagen molecule consists of three α chains, which can be either homotrimers or heterotrimers. As a significant constituent of the ECM, collagen I plays a vital role in various physiological processes, such as wound repair and organ development (30). Type V and XI collagens are involved in the formation and structural organization of collagen fibrils. Type V collagen is found in heterotypic fibrils alongside type I collagen, whereas type XI collagen is present in heterotypic fibrils alongside type II collagen. These collagens exert influence over the diameter and morphology of fibrils (31). The regulation of collagen fiber formation is mediated by small leucine-rich proteoglycans (SLRPs), a family of proteoglycans known to be involved in the assembly of fibrillar collagen. SLRPs possess a conserved carboxy terminus that contains leucine-rich repeats (LRRs), which in turn contain collagen-binding regions facilitating their interaction with collagen fibers. The pivotal role of SLRPs in governing multiple facets of collagen fiber formation, such as size, growth, shape, and content, is achieved through their interaction with collagen molecules and subsequent influence on their assembly (6). BM collagens are integral ECM proteins crucial for maintaining the structural integrity and functionality of basement membranes. The predominant constituent of basement membranes is collagen IV, which establishes a network within the membrane and engages in interactions with various proteins, including integrins, discoidin domain receptors (DDRs), and G protein-coupled receptors (32). Additionally, collagen VI, another significant basement membrane collagen, forms beaded microfibrils that serve as anchors between basement membranes and the interstitial matrix (33). Collagen VII is involved in dermal-epidermal adhesion and mediates the attachment of the epidermis to the basement membrane. Collagen VII is implicated in the adhesion between the dermis and epidermis, facilitating the attachment of the epidermis to the basement membrane (34). Collagen XVII, on the other hand, is a transmembrane collagen that contributes to cell-matrix interactions and is crucial for the attachment of epithelial cells to basement membranes (35). Mutations affecting collagen XVII can lead to autoimmune blistering diseases, such as Bullous Pemphigoid (BP) and linear IgA dermatosis (35). Consequently, these distinct classes of collagens possess unique structural and functional characteristics, and they play significant roles in the development, maintenance, and repair of tissues. The existence of different classes of collagens in the matrix of diverse tissues allows for the specialization and diversity of tissue functions (27).

Proteoglycans, which are a type of glycoproteins, consist of a core protein that is covalently attached to one or more glycosaminoglycan chains. This particular class of molecules can be classified into four main groups: Heparan sulfate proteoglycans (HSPGs), Chondroitin- and dermatan sulfate-containing proteoglycans (CSPGs and DSPGs), SLRPs, and Testican/SPOCK family. These proteoglycans are predominantly found in the extracellular matrix of connective tissues and play pivotal roles in various biological processes (36). Syndecans, a group of cell surface proteoglycans, exert significant influence on the development and progression of cancer. These proteoglycans consist of a core protein that is covalently linked to heparan sulfate (HS) chains. Syndecans have been observed to stimulate proliferative signals and enhance the survival of cancer cells (37). Perlecan, a multifunctional proteoglycan, assumes significant roles in diverse biological processes, such as blood vessel development and chondrogenesis. Within the context of blood vessel development, perlecan actively participates in the angiogenic process, facilitating the formation of new blood vessels. This is achieved through its interaction with fibroblast growth factor 2 (FGF2), an angiogenic factor, which triggers the binding and activation of perlecan, ultimately inducing capillary and vessel formation (38). Laminins, a group of expansive heterotrimeric multidomain proteins, hold significant importance in the composition of the extracellular matrix and exert influence over diverse cellular processes. Comprising three chains, namely α, β, and γ, these proteins exist in a minimum of 16 distinct isoforms within higher organisms. By activating signaling pathways via cell membrane receptors, laminins assume pivotal functions in differentiation, morphogenesis, and the development of organs (38). Similar to laminin, fibronectin (FN) is a glycoprotein that is widely present in the ECM. FN is released as a substantial dimeric glycoprotein, with the size of its subunits being subject to variation through alternative splicing. Its crucial functions encompass cell adhesion, migration, and differentiation, achieved by establishing connections with cells via integrins and other receptors (39). The process of FN polymerization is of utmost importance in the regulation of the composition and stability of extracellular matrix fibrils. It exerts control over the assembly and disassembly of the ECM as well as the adhesion sites between cells and the matrix. The maintenance of FN fibrils is contingent upon the polymerization of FN. This necessitates the binding of FN to integrin receptors, although integrin ligation alone does not adequately sustain FN matrix fibrils (40). FN plays a crucial role in the regulation of the ECM by facilitating the assembly of latent transforming growth factor-β (TGF-β) and latent TGF-β-binding protein-1 (LTBP1). Additionally, FN is also essential for the assembly of other ECM proteins, such as type I collagen, fibulin I, thrombospondin, and fibrinogen (41).

The interplay between proteoglycans and glycoproteins plays a crucial role in diverse biological phenomena, including the advancement of cancer. Specifically, in the context of cancer, hyaluronic acid (HA) emerges as a prominent proteoglycan constituent within the ECM, engaging in interactions with glycoproteins like CD44 and the receptor for hyaluronic acid-mediated motility (RHAMM) (42). The interaction between HA and CD44 expands the potential range of HA signaling in numerous biological and pathological processes, such as proliferative signaling, actin remodeling, cytoskeletal rearrangement, cell growth, and modulation of immune cells (43–48).

## Immune cells in the remodeling of the ECM

Multiple factors can mediate ECM remodeling. The roles of cancer-associated fibroblasts (CAFs), LOX, MMPs and other stromal cells or proteins in ECM remodeling have been well reviewed (49–55). In the following section, we focus in the roles of various immune cells in the ECM remodeling.

### Tumor-associated macrophages

The tumor-associated macrophages (TAMs) play a significant role in orchestrating the remodeling of the ECM. TAMs engage in a precise and intricate process of degrading existing ECM components while simultaneously stimulating fibroblasts and synthesizing new proteins. Consequently, this leads to the formation of a reactive stroma, which provides a conducive environment for tumor cell invasion, proliferation, and angiogenesis (56).

The regulation of TAMs within the tumor microenvironment TME involves intricate interactions and nuanced influences. Notably, the presence of macrophage colony-stimulating factor (M-CSF), secreted by the cancer cells themselves, is accompanied by a diverse range of chemokines and extracellular proteins. This collective assemblage of factors intricately modulates the functions of TAMs, thereby directing their crucial roles within the TME (57). TAMs demonstrate a notable adaptability in their function, conventionally associated with CAFs, in the process of collagen fibrillogenesis. They proficiently participate in the formation of the extracellular matrix by depositing, cross-linking, and aligning fibrillar collagens, thereby exerting their influence beyond immune responses to actively shape the structure of the tumor (57).

The significance of TAMs’ specialized function in breast cancer becomes evident through the secretion of MMP11, which plays a critical role in facilitating the migration of breast cancer cells that are HER2-positive. This mechanism, mediated by the CCL2-CCR2 signaling pathway, highlights the precise and selective actions of TAMs across various subtypes of cancer (58). TAMs exhibiting elevated levels of B7-H3 expression contribute an additional facet to the promotion of regulators involved in ECM construction and angiogenesis, including MMP2, VEGF-A, and TGF-β. These activities effectively facilitate the degradation of ECM and the development of novel vascular networks, crucial mechanisms in the metastasis of tumors (59).

Podoplanin-Expressing Macrophages (PoEMs), a distinct subset of TAMs, assume a crucial function in breast cancer progression by facilitating lymphangiogenesis and lymphoinvasion. The selective adherence of PoEMs to the walls of lymphatic vessels exemplifies the targeted and specialized nature of TAM interactions within the TME (60). The presence of DAB2 in TAMs in gastric cancer elucidates an additional aspect of their impact on the advancement and spread of tumors. The intricate involvement of DAB2-positive macrophages in haptotaxis, integrin recycling, and mechanotransduction underscores the complex nature of TAMs’ contribution to the field of cancer biology (61). The multifaceted functions of TAMs in ECM remodeling, targeted enzyme and growth factor secretion, as well as their intricate interactions with neighboring cells, underscore their pivotal role in the advancement and spread of cancer.

### Neutrophils

Neutrophils are involved in the process of ECM remodeling. Neutrophil extracellular traps (NETs), specifically through the actions of neutrophil elastase (NE) and matrix metalloproteinase 9 (MMP9), play a significant role in targeting laminin. The remodeling of laminin results in the generation of a novel epitope, which subsequently triggers integrin α3β1 signaling in cancer cells. This activation serves as a pivotal event in reactivating dormant cancer cells, initiating a cascade of biological processes that ultimately lead to tumor growth and metastasis (61). The activation of the integrin β1 outside-in signaling pathway, which is initiated by the NETs, plays a significant role in amplifying the aforementioned process. This activation leads to a cascade of cellular events involving key mediators such as FAK, ERK, myosin light chain kinase (MLCK), myosin light chain 2 (MLC2), and YAP. The combined action of these mediators is essential in promoting the proliferation and potential metastasis of previously quiescent cancer cells (62).

Cathepsin G is another important constituent of neutrophil serine proteases, released by activated neutrophils through azurophil granules (63). In previous research, through comparing the effect of treatment with cathepsin G and other proteases, including neutrophil elastase against FN coated substrates, cathepsin G is found to weaken adherence to culture substrates and induces E-cadherin-dependent aggregation of MCF-7 human breast cancer cells through its protease activity (64). This phenomenon for NETs-associated Cathepsin G is also observed in hepatocellular carcinoma (HCC). This investigation demonstrated that HCC cells-derived cytokine IL-8 triggered NETs formation in an NADPH oxidase-dependent manner, and NETs-associated cathepsin G promoted HCC metastasis *in vitro* as well as vivo (65).

### Dendritic cells

The dendritic cells (DCs) also play a role in the remodeling of the ECM. The interaction between ECM and the bone marrow-derived cells (BMDCs) exhibits distinct characteristics in the context of lung cancer. Tumor cells modify the genetic expression of BMDCs, resulting in heightened activation of hepatic stellate cells (HSCs) and subsequent synthesis of collagen type I. This increased activity not only remodels the ECM, but also establishes a favorable environment for the recruitment of granulocytic myeloid-derived suppressor cells (gMDSCs) to the liver. The presence of these cells in the liver promotes an immunosuppressive microenvironment, thereby facilitating the occurrence of liver metastasis (66).

Monocyte-derived dendritic cells play a crucial role in the remodeling of the ECM. The mechanism involves the expression and activation of heparanase, a heparan sulfate-degrading enzyme, by monocytes and early immature DCs. Heparanase is synthesized and retained in an active form in mature DCs. It accumulates in membrane extensions upon DC maturation, which allows for ECM degradation. The active heparanase in mature DCs enables the transmigration of DCs through the ECM. This transmigration is important for the migration of DCs from peripheral tissues to regional lymph nodes, where they present antigenic peptides to T lymphocytes. The presence of heparanase on the cell surface and in membrane extensions is involved in the migratory activity of mature DCs. The degradation of ECM heparan sulfate by heparanase facilitates DC migration and influences DC phenotype. The remodeling of the ECM by monocyte-derived DCs is essential for their functioning as antigen-presenting cells and their role in initiating immune responses (67).

### Natural killer cells

The involvement of natural killer (NK) cells, specifically through the NK cell receptor NKp46, introduces an additional level of intricacy in the regulation of the tumor microenvironment. Through the secretion of interferon-gamma (IFN-g), NK cells exert a substantial impact on tumor structure and the spread of cancer cells, thereby highlighting their indispensable contribution to the immune landscape within tumors (68).

Another mechanism that NK cells involve in ECM remodeling is the expression of heparanase. When NK cells are activated, they upregulate the transcription and protein levels of the heparanase gene. The enzymatic activity of heparanase allows NK cells to degrade the ECM, which is essential for cell invasion and migration across basement membranes. This process is facilitated by the ability of heparanase to break down HS chains in the ECM. Heparanase-deficient NK cells exhibit impaired invasion and migration capabilities, highlighting the importance of heparanase in these processes (69).

### T cells

T cells, particularly CD8+ T cells, play a crucial role in the remodeling of the ECM following chemotherapy. This is achieved through the direct influence of these cells on the collagen structure within the ECM, primarily by secreting LOX. Considering that B and T lymphocytes are significant contributors of LOX, their involvement in ECM remodeling assumes greater significance, thereby emphasizing their critical role in the overall context of tumor advancement and metastasis (70).

The intricate interplay between the ECM and immune cells constitutes a convoluted network that plays a pivotal role in the mechanisms underlying tumorigenesis, progression, and metastasis.

## ECM remodeling and immunosuppression in the TME

Tumor progression is characterized by extensive restructuring of the adjacent ECM, resulting in the development of a tumor-specific ECM that is frequently enriched in collagen and exhibits heightened rigidity. The interaction between immune cells and the ECM entails migratory mechanisms reliant on proteases and integrins within interstitial matrices. The traversal of basement membranes by immune cells entails receptor-mediated interactions and breaches induced by mechanical tension. The biochemical heterogeneity and spatial arrangement of ECM constituents significantly influence the migration, maturation, and activation of immune cells (71).

The intricate and ever-changing network, abundant in constituents such as collagen and fibronectin, functions not only as a structural framework but also plays a vital role in facilitating cellular signaling and modulating immune responses. An illustrative example of this is observed in the impact of the ECM on T cells, whose ability to perceive and react to environmental stiffness underscores their mechanosensitivity (72). T cell receptors (TCRs) exhibit catch bonds when subjected to tension, thereby augmenting activation and signaling. These bonds, bolstered by forces generated by cells, result in more robust interactions with antigen-presenting molecules, thereby influencing T cell discrimination between self and non-self peptides (73). The ECM component significantly impacts T cell dynamics, as evidenced by alterations in T cell behavior within dense collagen matrices, including a reduction in cytotoxic activity and an elevation in regulatory T cell markers (74). The orientation of collagen fibers has an impact on the distribution and movement of CD8 T cells within the tumor stroma (75). Likewise, the ECM has an effect on dendritic cells and macrophages. Substrate stiffness can induce a proinflammatory phenotype in dendritic cells through pathways such as transcriptional co-activator with PDZ-binding motif/yes-associated protein (TAZ/YAP) signaling, and it can also influence macrophage polarization, with higher stiffness favoring the M1 type and lower stiffness favoring the M2 type (76). Additionally, the ECM plays a role in immune cell adhesion and migration (71). An example of a crucial process in immuno-oncology involves the interaction between CD44 on activated T cells and immobilized HA in the ECM. This interaction plays a vital role in T cell rolling and adhesion, thereby facilitating their movement and function (77). Understanding such intricate interactions between ECM and immune cells, including their mechanical properties and signaling pathways offer insights into immuno-oncology.

### ECM and T cells migration and activation

**Figure 2:**
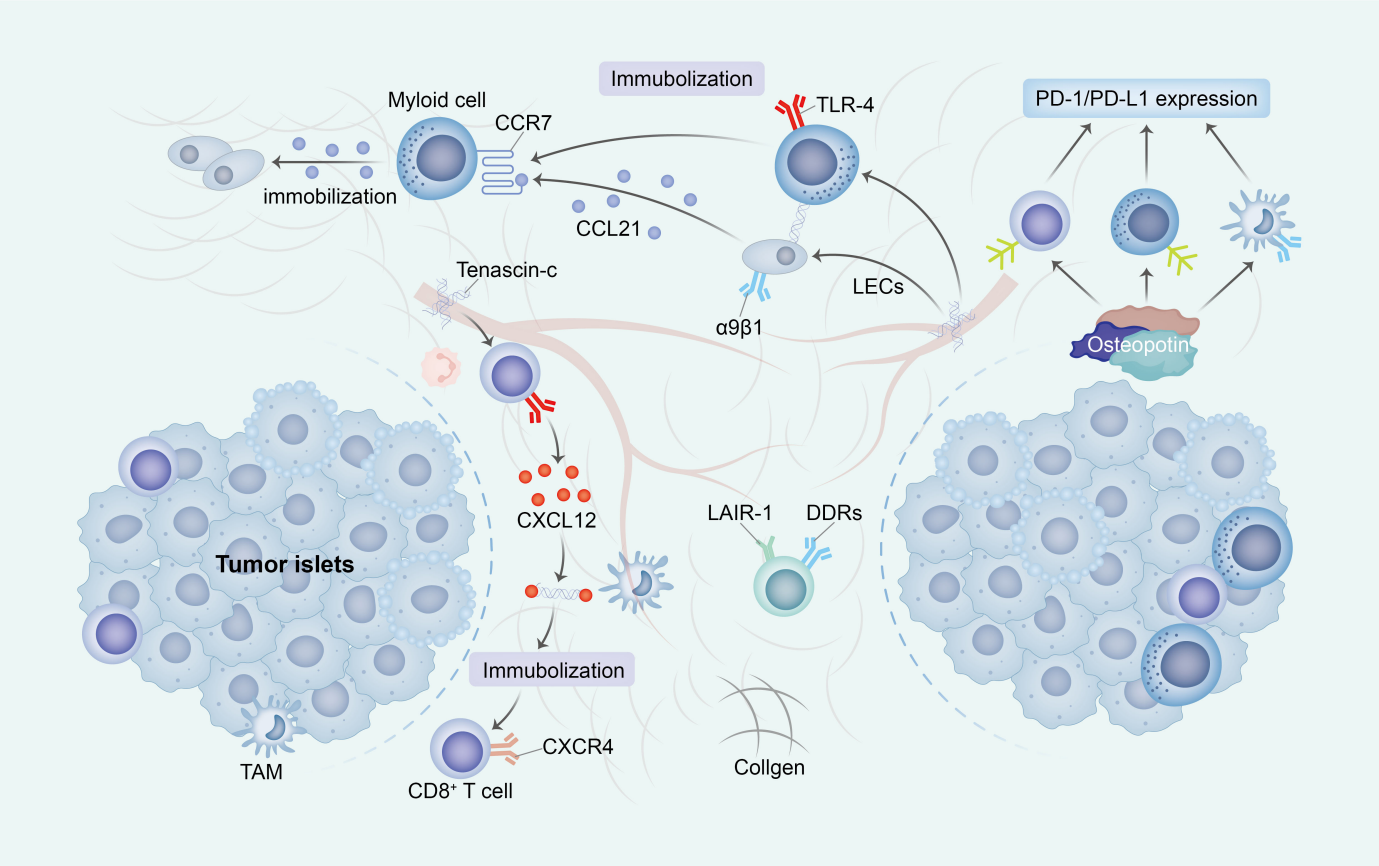
The ECM impedes the activation and migration of immune cells. The construction of an immunosuppressive TME through ECM change exhibits diverse mechanisms that promote immune escape. Primarily, the increased stiffness of ECM subsequently impede the migration of immune cells into tumor islets by establishing a physical barrier or directly inhibit cell-ECM surface interactions (through receptors such as DDRs and LAIR-1). Additionally, the presence of TNC immobilizes T cells alongside immunosuppressive cells and stromal cells, thereby entrapping immune cells within the ECM. Ultimately, OPN elicits the upregulation of PD-1/PD-L1 expression in immune cells, consequently leading to the induction of immunosuppression. ECM, extracellular matrix; TME, tumor micro environment; DDRs, discoidin domain receptors; LAIR-1, immunoglobulin-like receptor 1; TNC, Tenascin-C; OPN, Osteopontin.

The spatial organization of collagen fibers within the tumor microenvironment is a strategic configuration that exerts a profound impact on the functionality and conduct of immune cells, surpassing its mere physical obstructive role. The interconnection and alignment of collagen fibers result in a compact and inflexible framework enveloping tumor cells, characterized by a parallel arrangement of fibrils (78). Although this architecture does not entirely impede the initial infiltration of immune cells into the tumor tissue, it significantly impedes their subsequent migration towards the tumor core and critical structures such as islets (79) (**Figure 2**).

The presence of cross-linked collagen induces a significant physical impediment, impeding the mobility of immune cells by constraining the available space for migration and impeding their advancement. Additionally, the consistent arrangement of collagen fibers disrupts the customary adhesion mechanisms of immune cells, which typically rely on integrin receptors to navigate diverse ECM components. This unconventional topography obstructs the appropriate interaction of integrins, consequently disrupting the customary migratory signals (80).

In the present structured TME, the signaling pathways that are indispensable for guiding immune cells are also impacted. Conventionally, cells adhere to chemokines, which are chemical signals that steer their locomotion. Nevertheless, within a rigid, interlinked ECM, these signals may be unevenly dispersed or less readily attainable, thereby disorienting the immune cells and compromising their capacity to efficiently navigate towards their intended destinations (81).

**Figure 3:**
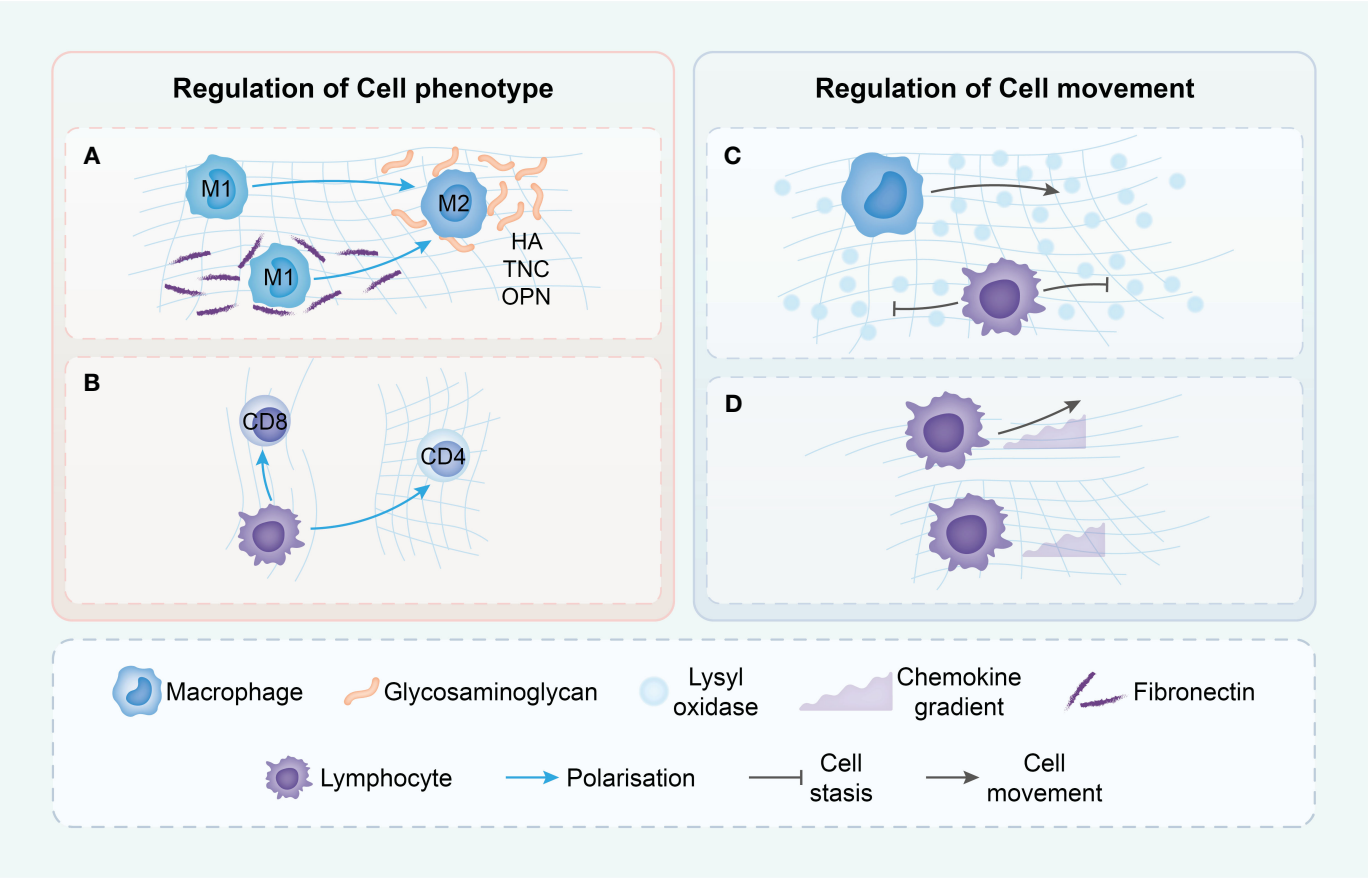
Proposed mechanisms though which the tumor ECM modulates immune cell phenotype and movement. **(A)** Macrophages in dense ECM demonstrate alternative states of activation dependent on the presence of various ECM components. **(B)** T cells phenotype is dependent upon ECM density with loose matrix supporting cytotoxic T cells but dense matrix leading to immune-inhibitory phenotypes. **(C)** T cells movement is driven by chemokine gradients in loose ECM, but in dense ECM, T cells do not demonstrate chemokine-directed movement. **(D)** ECM structure in TME may differentially modulate the migration of T cells and macrophages, with only macrophages able to move in dense, highly cross-linked matrices. HA, hyaluronic acid; TNC, Tenascin-C; OPN, Osteopontin.

The lymphoid cell dynamics and motility are greatly impacted by the ECM, specifically collagen types I and IV, and fibronectin. These components of the ECM play a crucial role in enhancing the movement of lymphoid cells, particularly in tissue microenvironments that exhibit diverse ECM compositions. Notably, T cells exhibit a preference for navigating through regions characterized by sparse collagen and fibronectin fibers, while actively avoiding denser matrices that are cross-linked by LOX (45) (**Figure 3**).

This preference is not without consequence; the direct impact of collagen fibers on cell motility extends to the cytoskeletal organization and overall shape of the immune cells (82). When placed in three-dimensional environments of varying collagen densities, T cells respond differently. In high-density collagen matrices, T cell proliferation is markedly reduced compared to low-density environments. This phenomenon is also reflected in the altered balance between CD4+ and CD8+ T cells under prolonged exposure to high-density collagen (83) (**Figure 3**).

In the realm of cancer, particularly in the case of human breast cancer, the collagen density present in the tumor microenvironment exhibits a direct association with the prevalence and functionality of T cells. Mammary tumors characterized by high collagen density demonstrate a decrease in the infiltration of CD8+ T cells, highlighting the influential role of collagen density in the dynamics of T cells. This correlation is further substantiated by the observation that a matrix with high density prompts a decrease in the expression of markers associated with cytotoxic activity in T cells, while concurrently upregulating markers associated with regulatory T cells (74). This transition not only impedes the survival of T cells but also promotes the prevalence of CD4+ T cells over CD8+ T cells, corroborating the hypothesis that tumor matrices selectively impede the functionality of cytotoxic CD8+ cells while preserving CD4+ cells. These CD4+ cells play a crucial role in sustaining an elevated inflammatory condition within tumors (74).

The orientation, spacing, and density of collagen fibers play a significant role in influencing the distribution and migration of CD8 T cells within the tumor stroma. Regions with lower collagen density exhibit a higher abundance of CD8 T cells, contrasting with areas featuring looser collagen (**Figure 3**). A negative correlation is observed between collagen density in specific stromal regions and the quantity of CD8 T cells present. Furthermore, in dense collagen regions, CD8 T cell movement is constrained compared to more porous areas, where enhanced mobility is observed (75). The meticulous examination highlights the substantial impact of the physical attributes of the ECM on the conduct of immune cells, particularly within the intricate and ever-changing milieu of tumors.

In the investigation of ovarian cancer, it is observed that the ECM, specifically its fibronectin fibers, assumes a central role in shaping immune responses. These fibers establish conduits that facilitate the random yet purposeful migration of T cells, particularly in areas where the matrix exhibits a loose organization. This spatial configuration is of utmost importance in directing T cell locomotion, thereby augmenting immune surveillance and bolstering antitumor immunity in such areas (79).

**Figure 4:**
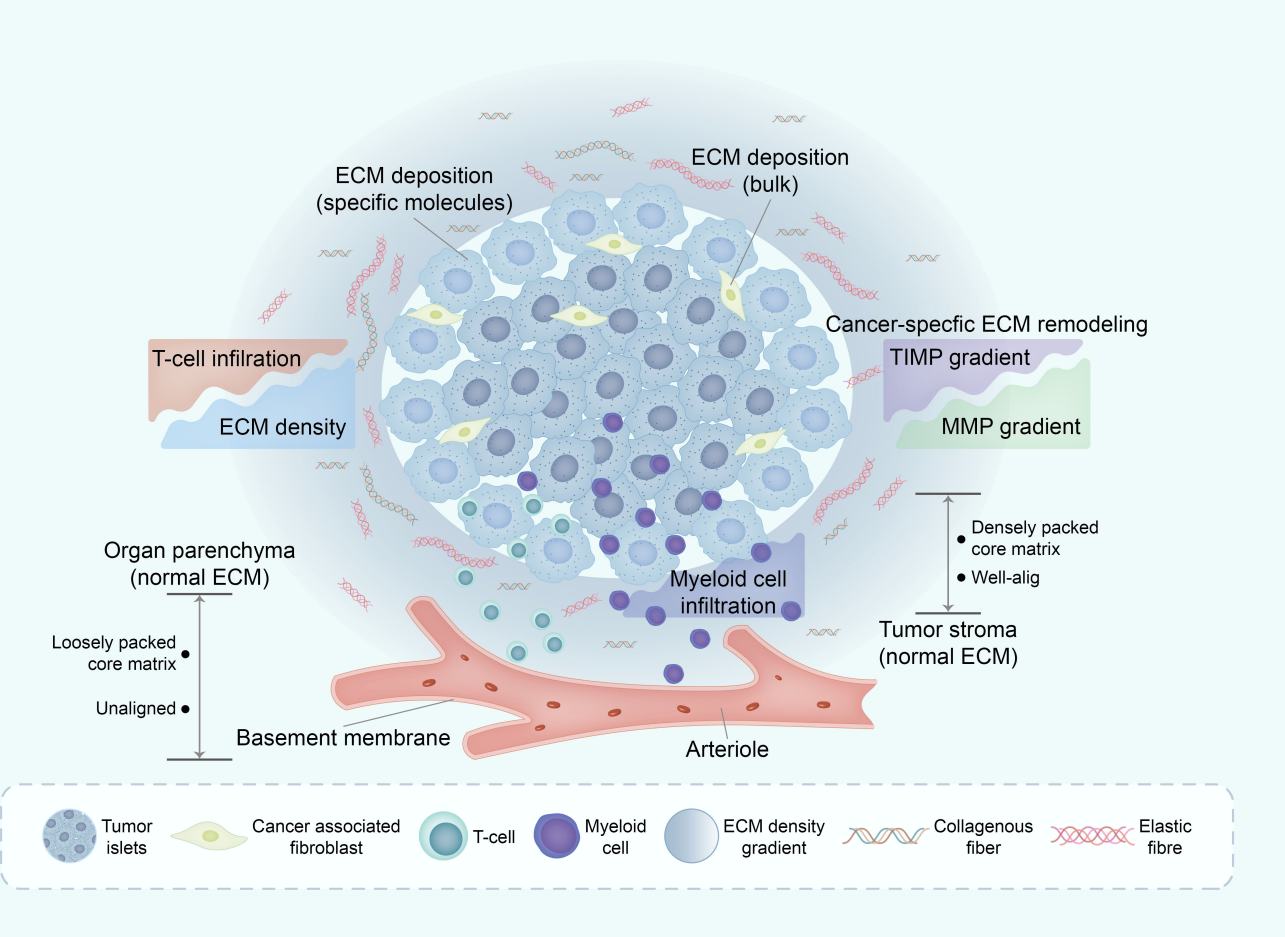
Deregulation of ECM in TME affects immune infiltration. The mechanisms underlying ECM remodeling in TME are complex, with the general line being the subtle balance between ECM-decomposing enzymes (mainly MMPs) and their corresponding inhibitors (mainly TIMPs). One of the major consequences of ECM remodeling in TME is collagen alignment, which partially regulates immune cell trafficking within the TME. Through this and other mechanisms, the ECM in TME excludes some immune cell subsets (such as infiltrating CD8+ T cells) whilst enabling active infiltration of others, such as macrophages. ECM, extracellular matrix; TME, tumor micro environment; MMPs, matrix metalloproteinases; TIMPs, tissues inhibitors of MMPs.

However, the situation contrasts in the case of pancreatic cancer. In this context, the presence of dense collagen networks within the tumor stroma presents a significant obstacle, impeding the efficient interaction between T cells and tumor cells. Consequently, the contact guidance mechanism of the ECM assumes a dominant role in this environment, leading to the accumulation of T cells within the stroma. This accumulation effectively supersedes the chemokine-mediated migration that typically guides T cells within the tumor. Consequently, when activated T cells exit the bloodstream and encounter the dense matrix of the pancreatic stroma, they transition from chemokine-guided migration to matrix-guided movement. This shift in migration pattern often leads to the misdirection of T cells, steering them away from tumor cells (84) (**Figure 4**).

The divergent influence of the ECM on various cancer types underscores its substantial involvement in modulating immune cell behavior. In the context of pancreatic cancer, the compact collagen matrix not only hampers T cell infiltration into tumor cells but also exerts a profound effect on the functionality of tumor-infiltrating T cells. The elevated density of collagen within the tumor microenvironment can induce immunosuppressive effects. T cells cultured within high-density collagen matrices exhibit diminished cytotoxic activity and reduced secretion of IFN-γ, a pivotal cytokine crucial for mounting an effective immune response (74).

Furthermore, the mechanical characteristics of the ECM play a significant role in modulating T cell activation and proliferation in ovarian and pancreatic cancers (85). Specifically, the restructuring of the ECM, particularly in terms of its density and fiber alignment, directs the migration of T lymphocytes and influences their functional attributes. Consequently, these ECM properties have the potential to hinder T cell activation, expansion, and cytotoxic functions, thereby inducing immunosuppression within the tumor microenvironment (86).

### The influence of ECM stiffness on the APCs

**Figure 5:**
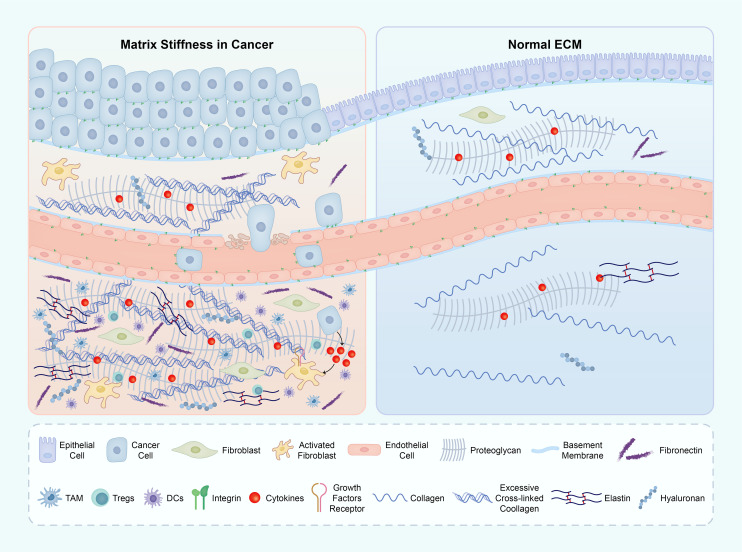
The ECM components in both the tumor microenvironment (TME) and normal tissue. The primary factor contributing to matrix stiffness in the TME is the excessive presence of collagen and HA. This heightened stiffness of the ECM significantly impacts various crucial biological processes throughout tumor development. The abbreviations used are ECM for extracellular matrix, TME for tumor microenvironment, and HA for hyaluronic acid. ECM, extracellular matrix; TME, tumor micro environment; HA, hyaluronic acid.

The complex structural modifications occurring within the ECM have a profound impact on the behavior of APCs, specifically macrophages and DCs, thereby exerting influence on immune responses within the TME (87) (**Figure 5**).

In macrophages, an increased stiffness of the matrix leads to a significant augmentation in M2 polarization and Lysyl oxidase-like 2 (LOXL2) expression. This effect is primarily mediated through the integrin β5-FAK-MEK1/2-ERK1/2 signaling pathway, which triggers an upregulation of hypoxia-inducible factor 1-alpha (HIF-1α), subsequently enhancing LOXL2 expression. Furthermore, the polarization state of M2 macrophages and the expression of LOXL2 in HCC tissues with COL1^High^/LOX^High^ provided further evidence of the regulatory role of matrix stiffness in macrophage polarization and LOXL2 expression (88).

Simultaneously, the stiffness of the substrate is of utmost importance in regulating the generation of reactive oxygen species (ROS) in macrophages. This regulation is accomplished by modifying focal FAK signaling, cytoskeletal arrangement, and the functionality of ROS-producing enzymes. It is worth noting that macrophages cultured on more pliable substrates exhibit heightened ROS production, inflammasome assembly, and nitric oxide (NO) discharge when exposed to lipopolysaccharide (LPS)/ATP stimulation, thereby favoring a proinflammatory M1 phenotype (89). Heightened matrix rigidity promotes the polarization of macrophages towards the M2 phenotype, which is frequently associated with tissue regeneration and immunosuppressive properties.

The highly expressed ECM component, HA, has also been found to bind to CD44 and initiate macrophage polarization towards the M2 phenotype (43). The interaction between HA and CD44 triggers downstream signaling pathways, including PI3K/Akt and MAPK, which subsequently lead to the expression of M2-associated genes and the secretion of M2-associated cytokines. On the other hand, downregulation or inhibition of HA and CD44 expression has been found to promote M1 polarization, which is associated with pro-inflammatory and anti-tumor activities. Suppression of the HA pathway has been shown to enhance M1 polarization and promote anti-tumor immune responses. Overall, HA-CD44 interaction can promote M2 polarization, while suppression of HA and CD44 can enhance M1 polarization (90).

In colon cancer cells, the influence of mechanotransduction mediator on macrophage polarization is observed in a co-culture model of THP-1 through genetic or pharmacologic inhibition of YAP. Inhibition of YAP solely in differentiating THP-1 cells resulted in reduced of M2 polarization, indicating a potential anti-inflammatory function of YAP in this context (91).

The stiffness of the ECM in DCs is equally influential, as it impacts the expression of C-type lectin receptors (CLRs) that are crucial for antigen internalization, integrin-dependent migration, and the initiation of immune responses. DCs cultured on stiffer substrates (12 kPa) exhibit decreased expression of CLRs, including the Macrophage Mannose Receptor (MMR, CD206) and DC-SIGN (CD209), in comparison to those cultured on softer substrates (2 kPa) (92). Moreover, the utilization of more rigid substrates induces a proinflammatory phenotype in DCs by triggering the activation of diverse intracellular pathways such as TAZ/YAP, calcium signaling, and the mTOR pathway. This activation leads to an increased production of inflammatory cytokines and an augmented expression of pivotal surface molecules that are essential for the proper functioning of DCs. Additionally, it stimulates the metabolic activity of DCs, particularly impacting glycolysis and the pentose phosphate pathway (93).

Recent research has elucidated the significant influence of ECM stiffness on regulatory immune cells, particularly highlighting the involvement of specific proteins and cell types. Notably, the protein talin plays a central role in this context, as it is indispensable for the preservation of regulatory T (Treg) cells. The absence of talin in Treg cells has been observed to disturb their homeostasis and viability, thereby emphasizing its critical function in maintaining the stability and immunosuppressive characteristics of these cells (94).

In conjunction with the revelation regarding talin, the leukocyte-associated immunoglobulin-like receptor 2 (LAIR2) has been recognized as a pivotal facilitator of tumor cell infiltration. The noteworthy role of LAIR2 lies in its ability to augment the adherence of tumor cells to collagen I, a substantial constituent of the ECM. This mechanism assumes particular significance in the context of lung adenocarcinoma, wherein elevated LAIR2 expression in tumor-associated Treg cells correlates with heightened tumor advancement and metastasis (95).

The interplay between CAFs and Treg cells serves as a prime illustration of the intricate dynamics within the tumor microenvironment. It has been demonstrated that CAFs have the ability to induce differentiation in CD73+ γδ Treg cells (gdTregs), subsequently leading to the stimulation of interleukin 6 (IL6) secretion by CAFs via the adenosine/A2BR/p38MAPK pathway. This reciprocal interaction establishes a reinforcing feedback loop that holds significant importance in immune suppression and the advancement of breast cancer. Furthermore, it has been observed that CAF-S1 fibroblasts possess the ability to attract and retain T lymphocytes through the utilization of specific molecules, thereby facilitating their survival and promoting their differentiation into Tregs (96). These discoveries collectively underscore the existence of a complex network of interactions involving the ECM, regulatory immune cells, and CAFs (**Figure 5**).

### The interaction between receptors and ECM components: DDRs and LAIRs

The engagement of the DDRs with collagen, a fundamental constituent of the extracellular matrix, serves to facilitate a diverse range of cellular responses. DDR1, functioning as a receptor tyrosine kinase, plays a pivotal role in leukocyte morphogenesis and function, particularly in their migration through collagen-rich environments. This migratory process is regulated by the DDRs’ capacity to recognize and bind to specific collagen motifs, thereby initiating intracellular signaling cascades that orchestrate the reorganization of the cytoskeleton, a critical mechanism for cellular locomotion. Notably, the overexpression of DDR1a in THP-1 cells significantly enhances leukocyte migration, indicating its potential in promoting immune cell activity. On the other hand, the overexpression of DDR1b hinders the migration of leukocytes, indicating a multifaceted regulatory mechanism within the DDR1 family and emphasizing the significance of DDRs in precisely adjusting immune responses (97).

Further elucidating DDR1’s involvement in immune responses, its activation in macrophages upregulates proinflammatory mediators such as IL-1β, IL-8, MIP-1α, and MCP-1. This activation is facilitated through the p38 MAPK and NF-κB signaling pathways, underscoring the intricate participation of this receptor in inflammatory responses (98).

DDR2, a member of the DDR family, plays a significant role in the regulation of neutrophil chemotaxis within three-dimensional collagen matrices. Activation of DDR2 triggers the secretion of MMP, which is essential for the generation of chemotactic signals. This mechanism highlights the importance of DDR2 in guiding immune cells within the intricate tumor microenvironment (99).

Furthermore, the inhibition of DDR2 in conjunction with anti-PD-1 therapy has been observed to synergistically control tumor growth and metastasis in both cell line and mouse model studies. Consequently, DDR2 emerges as a promising target in the field of cancer immunotherapy. This understanding establishes a connection between DDR2’s physiological function in cellular signaling (100).

DDR1 also plays a role in the modulation of tumor immune dynamics through the facilitation of immunosuppressive factor secretion, such as TGF-β, upon activation by collagen fibers. This mechanism presents a promising avenue for therapeutic intervention, wherein the targeting of DDR1 or the manipulation of collagen fiber alignment may disrupt immunosuppression and bolster immune infiltration within tumors (101).

The presence of LAIR-1, a cell surface receptor found on different immune cells, contributes to the intricate immune regulation occurring within the tumor microenvironment. LAIR-1 exerts control over the activation of immune cells by interacting with collagen, resulting in the suppression of immune cell activation pathways (102). This phenomenon is particularly notable in chronic lymphocytic leukemia (CLL), as the expression of LAIR-1 is closely associated with the severity of the disease (103, 104).

The high expression of LAIR-1 in hepatocellular carcinoma (HCC) and oral squamous cell carcinoma (OSCC) is correlated with poor differentiation and the presence of an immunosuppressive environment, suggesting its involvement in immune evasion. This trend is similarly observed in breast carcinoma, where elevated levels of LAIR-1 are associated with more advanced disease stages (105, 106).

Moreover, the significance of LAIR-1 in immune suppression is emphasized by its presence on dendritic cells and macrophages, which impacts the immune response of tumor-specific T cells. The interaction between LAIR-1 and collagen, followed by signaling in CD8+ T cells, emphasizes its involvement in promoting T cell exhaustion and enhancing the expression of immune checkpoint molecules such as PD-1 and PD-L1, thereby facilitating the evasion of the immune system by tumors (107–109).

MMP-generated collagen fragments have been found to suppress immune responses through the activation of LAIR-1 (83). However, this effect can be counteracted by the LAIR-2-Fc fusion protein, which inhibits the interaction between LAIR-1 and collagen, thereby promoting T cell activity and antitumor responses (110). In the context of colorectal tumors, the combination of the LAIR-1 inhibitor NC410 and bintrafusp alfa exhibits potential in modulating macrophage profiles and enhancing T cell activation, suggesting a promising therapeutic approach (111).

In an alternative investigation, it has been demonstrated that LAIR-1 exerts a suppressive effect on cellular growth by impeding the phosphorylation of AKT and mTOR pathways, thereby diminishing the proliferation and migration of ovarian cancer cells. These findings imply that the principal function of LAIR-1 lies in the modulation of immune cells rather than exerting a direct impact on tumor cells (112).

### The immuno-oncological regulators in ECM: Tenascin-C and Osteopontin

Tenascin-C (TNC), an intricate protein found in the extracellular matrix, has been identified as a crucial regulator within the TME. Its multifaceted nature allows it to exert influence over numerous facets of tumor dynamics and interactions with the immune system (113).

The pivotal role of TNC lies in its influence on both tumor cells and the adjacent stroma. Notably, it diminishes the adhesion capabilities of cancer cells, thereby promoting their heightened ability to proliferate, migrate, invade, disseminate, and home. This decrease in adhesion, coupled with augmented cellular mobility, serves as a distinctive characteristic of aggressive tumor behavior, establishing a direct association between TNC expression and the potential for metastasis. This connection is particularly conspicuous in malignancies such as oral squamous cell carcinoma and breast cancer (114, 115).

Furthermore, TNC plays a crucial role in shaping the tumor microenvironment. It hinders the attachment and activation of CAFs and tumor-infiltrating lymphocytes (TILs), thereby impeding their growth and cytokine synthesis. This inhibitory mechanism serves as a strategic evasion strategy employed by tumors to evade immune surveillance and response, underscoring the significance of TNC in establishing an immune-suppressive microenvironment (113).

In addition to its immune-modulatory effects, TNC has demonstrated the ability to impede T-cell proliferation and reduce the secretion of vital cytokines, including IFN-γ. This inhibitory action also extends to T-cell migration within glioblastoma, where elevated levels of TNC hinder T-cell movement through the tumor’s extracellular matrix. This hindrance is attributed to the delayed activation of signaling pathways crucial for T-cell activation and mobility, such as ERK activation. Furthermore, TNC affects the cellular structure by inducing alterations in F-actin localization and the formation of podia-like protrusions in T cells (116, 117) (**Figure 2**).

The influence of TNC extends to the behavior of macrophages within the tumor microenvironment, specifically promoting their polarization towards an M2 phenotype that is immune-suppressive. This polarization is characterized by an increase in CD206 expression and a decrease in MHC surface expression, which further strengthens the tumor’s ability to evade the immune system. In the case of glioblastoma, TNC’s interaction with macrophages, particularly in the absence of CD47, enhances the production of pro-inflammatory factors and recruits TAMs, thus highlighting TNC’s role in manipulating the recruitment and function of immune cells (118, 119).

In the context of oral squamous cell carcinoma, TNC plays a crucial role in regulating the tumor microenvironment through its influence on lymphatic signaling. Specifically, it stimulates the production of CCL21 in lymphatic endothelial cells and promotes the proliferation of fibroblastic reticular-like cells. These actions collectively establish a favorable milieu for tumor expansion and immune suppression. Consequently, this manipulation leads to a disrupted CCL21 gradient, exacerbating the impairment of immune cell migration and compromising adaptive immunity (120).

The interaction between TNC and CXCL12 in the immobilization of T lymphocytes, particularly CD8+ T cells, presents an additional aspect of its immunosuppressive function. Through its binding to CXCL12, TNC generates an adhesive substrate that diminishes the motility of T cells, a crucial determinant for a proficient immune response against tumors (121) (**Figure 2**).

The therapeutic implications of autophagy in the dynamics of TNC are noteworthy. Specifically, the degradation of TNC, facilitated by Atg5 and Atg7, plays a pivotal role in triple-negative breast cancer (TNBC) tumors, as it promotes T cell infiltration and activity. Consequently, this presents a promising opportunity for intervention in the progression of TNC-mediated tumors and evasion of the immune system (122).

Moreover, the multifaceted roles of autophagy, encompassing the regulation of tumor cell behavior and modulation of immune cell function, highlight its significance in the intricate interplay between the ECM and the immune system.

Osteopontin (OPN), a multifunctional extracellular matrix protein, exerts a significant influence on immune regulation in diverse cancer types, intricately modulating the interplay between tumor progression and immune response (123).

The central aspect of OPN’s functionality lies in its expression by a variety of cells, such as macrophages, dendritic cells, and activated T cells. In addition to its structural role in the extracellular matrix, OPN plays a crucial role in facilitating prompt stress responses, immune stimulation, and tissue remodeling, highlighting its multifaceted nature (124–126).

The role of OPN in breast cancer is of significant importance, as it promotes anchorage independence by being secreted by cancer cells. This process is primarily facilitated through its interactions with cell surface receptors, namely integrin αVβ3 and CD44v3-6. These interactions enable breast cancer cells to detach from the extracellular matrix and proliferate autonomously, which is a crucial stage in tumor metastasis. The various OPN splice variants, namely OPN-a, OPN-b, and OPN-c, exhibit distinct functionalities in tumor formation, immune cell activation, and cytokine regulation (123).

The impact of OPN extends to various cancer types, demonstrating its multifaceted pathological functions. For instance, within the realm of extramedullary myelopoiesis and immune evasion, OPN stimulates the proliferation of myeloid progenitor cells in the spleen. This phenomenon is facilitated by the upregulation of CD44, acting as a receptor for OPN, thereby promoting the expansion of cell populations that facilitate tumor progression and immune resistance (127).

In the context of Helicobacter pylori-induced gastric cancer, the lack of OPN assumes a defensive function by mitigating chronic gastritis and safeguarding tumor cells against apoptosis mediated by inducible nitric oxide synthase (iNOS). This underscores the significance of OPN in promoting tumor advancement via inflammatory pathways (128).

Additionally demonstrating the influence of OPN on immune regulation, its presence in NSCLC is associated with the amplification of PD-L1 via the NF-κB pathway, which is utilized by tumor-associated macrophages. This amplification serves as a tactical maneuver employed by the tumor to elude immune surveillance and annihilation (129).

In the context of glioblastoma, the function of OPN undergoes a transition towards facilitating the infiltration of macrophages. OPN is expressed in both glioblastoma stem cells and different types of macrophages, acting as a chemoattractant that attracts macrophages into the tumor microenvironment. This infiltration plays a crucial role in establishing a conducive milieu for tumor expansion and immune suppression (130).

The involvement of OPN in colon cancer serves as a notable illustration of its ability to regulate immune responses. The absence of IRF8 results in elevated levels of CD44+ CD8+ T cells, yet the simultaneous elevation of OPN expression hampers their activation. Consequently, OPN functions as an immune checkpoint, impeding the activation of CD8+ T cells and fostering immune tolerance towards tumors (131).

In the context of hepatocellular carcinoma, the impact of OPN is evident in its capacity to induce the migration and polarization of macrophages towards an M2 phenotype, characterized by immunosuppressive properties. This effect is achieved through the activation of the CSF1-CSF1R pathway and the PI3K/AKT/NF-κB/p65 pathway, which collectively direct macrophages towards a state that promotes tumor proliferation and evades immune surveillance (132).

Pancreatic cancer unveils an additional facet of OPN’s functionality, as it engages in an interaction with the WDR5 protein and histone methyltransferases. This interaction disrupts the deposition of H3K4me3, resulting in the upregulation of genes associated with ECM-receptor interaction and facilitating immune suppression. Consequently, this mechanism assists in the evasion of the immune system by the cancer cells (133).

The diverse functions of OPN in various cancer types highlight its pivotal role in the convergence of tumor biology and immune regulation (134).

## Conclusions and perspectives

The investigation of the ECM within the TME has revealed a multifaceted and intricate landscape, distinguished by its complex structure and notable heterogeneity. This dynamic and diverse matrix surpasses its conventional function as a structural scaffold, as it not only facilitates cell migration, tissue growth, and organ function, but also serves as a physical barrier, an anchor, and a signal transducer (135). In the context of cancer, the dense ECM plays a substantial role in the advancement of the disease, as it disrupts cell polarity and adhesion, hyperactivates growth factor signaling, and promotes cancer progression. Tumors additionally contribute to this process of tissue remodeling through the activation of stromal cells, resulting in an augmented synthesis of the ECM constituents such as collagen and fibronectin (136). Consequently, these ECM components play a pivotal role in promoting the advancement of cancer.

It is imperative to confront the obstacles presented by the inherent heterogeneity of the ECM in solid tumors in order to make progress in cancer therapies. A promising and emerging approach involves the integration of anti-cancer therapies with interventions that specifically target the ECM. This strategy aims to disrupt the ECM’s protective function surrounding malignancies, thereby augmenting the effectiveness of conventional cancer treatments (137). Presently, therapeutic endeavors primarily concentrate on the selective targeting of ECM constituents, including collagens and fibronectin (138). These methods are directed towards either the constituents themselves or the mechanisms that govern their generation, encompassing pivotal enzymes and ECM producer cells such as CAFs.

Novel amalgamations of therapies, such as the integration of ECM-targeting techniques with immune checkpoint inhibitors or PI3K signaling inhibitors within nano-system carriers, exhibit potential (139). These amalgamations have the potential to augment treatment effectiveness while mitigating unintended toxicities. Considering the distinct ECM composition in various organs, customized strategies for each cancer type are imperative to achieve optimal outcomes.

Gaining a more profound understanding of the underlying mechanisms of ECM involvement in cancer progression holds the potential to unveil novel avenues for cancer control. As therapeutic modalities advance, embracing a comprehensive approach that encompasses the multifaceted functions of the ECM may revolutionize the field of cancer therapy, ultimately leading to improved prognostic outcomes for individuals affected by the disease.

## Author contributions

WD: Writing – review & editing, Methodology, Project administration, Writing – original draft. XX: Writing – original draft, Investigation. FH: Writing – original draft, Formal analysis, Visualization. JY: Conceptualization, Funding acquisition, Supervision, Writing – review & editing.
